# Draft genome sequence of *Proteus sp*. strain NGCRVN-01, isolated from a tertiary care hospital in Bangladesh

**DOI:** 10.1128/mra.00175-25

**Published:** 2025-06-17

**Authors:** Fahad Khan, Katha Saha, Md. Mashiur Rahaman, Abdus Sadique, Monsur Murshid, Jahidul Alam, Fariza Shams, Md. Mainul Hossain, Kamruzzaman Rumman, Maqsud Hossain

**Affiliations:** 1 NSU Genome Research Institute (NGRI), North South University 54495, Dhaka, Dhaka Division, Bangladesh; 2 Department of Biochemistry and Microbiology, School of Health and Life Sciences, North South University 54495, Dhaka, Bangladesh; 3 National Institute of Cancer Research and Hospital (NICRH) 332897, Dhaka, Bangladesh; Queens College Department of Biology, Flushing, New york, USA

**Keywords:** *Proteus sp.*, antimicrobial resistance gene, hospital-acquired infections (HAIs)

## Abstract

We report the draft genome sequence of *Proteus sp*. strain NGCRVN-01, isolated from a tertiary care hospital in Bangladesh. The 4.2 Mb genome revealed multiple antimicrobial resistance genes, indicating its potential role in hospital-acquired infections and its capacity to acquire and disseminate resistance determinants in clinical environments.

## ANNOUNCEMENT

As part of the normal gut flora, *Proteus* spp., belonging to the order *Enterobacterales* and family *Morganellaceae*, can act as an opportunistic pathogens, particularly in nosocomial settings (1). *Proteus spp*. are the third leading cause of hospital-acquired infections (HAIs) (2), with a higher burden in low- and middle-income countries, where up to 25% of hospitalized patients are affected (3, 4).

*Proteus sp*. strain NGCRVN-01 was isolated from a freshly collected stool sample of a diarrheal patient at Dhaka International Medical College and Hospital, Bangladesh (23°46'26.3"N 90°21'41.5"E), on 16 December 2022. The sample was collected in sterile stool collection containers and transported on ice to the laboratory within 2 hours of collection. For enrichment, 1 mL of stool suspension was inoculated into 9 mL of Luria-Bertani (LB) broth (HiMedia, India) and incubated aerobically at 37°C for 6 hours. Following enrichment, a loopful was streaked on MacConkey agar (Oxoid, UK) and incubated aerobically at 37°C for 24 hours. Pale, non-lactose fermenting colonies were selected and subcultured for purity. Biochemical identification was carried out using IMViC tests, urease, and Triple Sugar Iron slant reactions, following standard microbiological protocols (5).

Genomic DNA was extracted from a single colony grown overnight in LB broth at 37°C using the QIAamp DNA Mini Kit (QIAGEN, Germany). Sequencing libraries were prepared with the Illumina Nextera XT Kit and sequenced on the Illumina MiSeq platform (2 × 150 bp) at the NSU Genome Research Institute, North South University. A total of 778,024 paired-end reads were generated. Quality assessment was performed using FastQC v0.12.1 (6), and *de novo* assembly was conducted with Unicycler (Version: November 2024) (7), producing a total genome size of 4,224,718 bp with a GC content of 38%. The assembly resulted in 96 contigs with an N50 value of 185,726 bp. Genome annotation was performed using the NCBI Prokaryotic Genome Annotation Pipeline v6.9. The annotation identified 4,047 genes, including 3,915 protein-coding genes, 3 rRNAs, 81 tRNAs, 1 mRNA, and 42 pseudogenes. The CheckM v1.2.3 analysis estimated a genome completeness of 88.26%.

The antibiotic resistance gene profile of *Proteus* sp. strain NGCRVN-01 was predicted using ResFinder v4.6.0 (8), applying a threshold of 90% sequence identity and 60% minimum gene coverage. Detected resistance genes included *aph*(6) (aminoglycoside and streptomycin), *qnrD1* (fluoroquinolone and ciprofloxacin), *hugA* (beta-lactam), *sul2* and *dfrA1* (folate pathway inhibitors: sulfamethoxazole and trimethoprim), *mph(F*) and *msr(E)* (macrolide), *msr(E)* (streptogramin B), *tet(B)* (tetracycline), and *floR* (amphenicol). While these gene calls are based on sequence similarity, they were consistent with phenotypic resistance patterns, supporting the strain’s clinical significance as a multidrug-resistant pathogen associated with HAIs.

The genome of *Proteus sp*. NGCRVN-01 harbors the plasmid-encoded *ipaH* gene, indicating invasive potential akin to Shigella and enteroinvasive *Escherichia coli* (9). High-confidence BLAST hits (VFDB, November 2024) (10) also revealed motility and metabolic genes (*cheA*, *cheZ*, *flgG*, and *fliA*), supporting environmental adaptability and host colonization. The presence of multiple virulence factors highlights this strain’s clinical relevance as a multidrug-resistant, potentially invasive pathogen.

## Data Availability

The whole-genome shotgun project has been deposited at DDBJ/ENA/GenBank under the accession number JBJHQC000000000. Raw sequencing reads are available in the NCBI Sequence Read Archive (SRA) under accession number PRJNA1185919. The associated BioProject and BioSample records are PRJNA1185919 and SAMN44732074, respectively. Additionally, the ResFinder analysis output files have been made publicly accessible via Figshare at https://doi.org/10.6084/m9.figshare.28443650.v1.
